# A rare case of ectopic ACTH syndrome with rhabdomyolysis

**DOI:** 10.1186/s12902-021-00755-0

**Published:** 2021-05-10

**Authors:** Wei Qiang, Sucai Song, Tianjun Chen, Zhe Wang, Jun Feng, Jiaojiao Zhang, Hui Guo

**Affiliations:** 1 Department of Endocrinology and Metabolism, The First Affiliated Hospital of Xi’an Jiaotong University, No.277 West Yanta Road, 710061 Xi’an, People’s Republic of China; 2 Department of Endocrinology, The First Affiliated Hospital of Zhengzhou University, 450052 Zhengzhou, People’s Republic of China; 3 Department of Respiratory and Critical Medicine, The First Affiliated Hospital of Xi’an Jiaotong University, 710061 Xi’an, People’s Republic of China; 4 Department of Thoracic Surgery, The First Affiliated Hospital of Xi’an Jiaotong University, 710061 Xi’an, People’s Republic of China; 5 Department of Vascular Surgery, The First Affiliated Hospital of Xi’an Jiaotong University, 710061 Xi’an, People’s Republic of China; 6 Department of Pathology, The First Affiliated Hospital of Xi’an Jiaotong University, 710061 Xi’an, People’s Republic of China

**Keywords:** Cushing’s syndrome, Ectopic ACTH syndrome, Hypokalaemia, Rhabdomyolysis

## Abstract

**Background:**

Manifestations of hypokalaemia in ectopic adrenocorticotropic hormonesyndrome(EAS) vary from mild muscle weakness to life-threatening arrhythmia. Herein, we present a rare case of EAS with concomitant rhabdomyolysis(RM) as a result of intractable hypokalaemia.

**Case presentation:**

A 64-year-old man was admitted for limb weakness and facial hyperpigmentation for 2 weeks. Lab tests revealed intractable hypokalaemia (lowest at 1.8 mmol/L) and metabolic alkalosis. The diagnosis of RM was based on a creatine kinase(CK)level of 5 times the upper limit. The elevated CK and myohemoglobin (Mb) levels returned to within the normal range after the alleviation of hypokalaemia. The patient was diagnosed with ACTH-dependent Cushing’s syndrome (CS) based on unsuppressed serum cortisol after a low-dose dexamethasone suppression test(LDDST) and remarkably elevated ACTH levels. The diagnosis of EAS was made based on the results of a high-dose dexamethasone suppression test(HDDST) and bilateral inferior petrosal sinus sampling(BIPSS). Multiple lymph nodes in the left supraclavicular fossa, right root of neck, mediastinum and bilateral hili of the lung were found with abnormal uptake of ^68^Ga-DOTA-NOC. Mediastinoscopic lymph node biopsy was performed. The pathological diagnosis was small-cell and large-cell neuroendocrine carcinoma with positive ACTH staining. The patient was prescribed mifepristone and received one cycle of chemotherapy. The patient could not tolerate subsequent chemotherapy and died of dyscrasia.

**Conclusions:**

RM is a rare complication of EAS with insidious onset and atypical clinical manifestations. Serum potassium levels should be vigilantly monitored to avoid RM in EAS.

## Background

Cushing’s syndrome (CS) is a set of clinical syndromes caused by excessive production of cortisol. Ectopic adrenocorticotropic hormone syndrome (EAS) accounts for approximately 12-17 % of CS cases in which ACTH is produced by tumours [1, 2]. Diagnosis and treatment are more challenging for EAS than for other types of CS. The classical features of CS, such as moon face, plethora and central obesity, are absent in patients with aggressive tumours of short duration and cachexia. The localization of the tumours is sometimes time consuming or ultimately futile, even when functional imaging is available. Additionally, extreme hypercortisolism results in a high risk of life-threatening opportunistic infections and overwhelming biochemical disorders, among which hypokalaemia is the most prominent. The manifestations of hypokalaemia vary greatly from mild muscle weakness to life-threatening arrhythmia. Herein, we present a rare case of EAS with concomitant rhabdomyolysis(RM) resulting from intractable hypokalaemia.

## Case presentation

A 64-year-old man was admitted to a local hospital for limb weakness and facial hyperpigmentation for 2 weeks. He also reported oedema of the lower limbs for 1 week. He had a history of smoking for 40 years(30 cigarettes per day). Lab tests revealed severe hypokalaemia (1.8 mmol/L, normal range 3.5–5.5 mmol/L) and metabolic alkalosis. Potassium supplementation (KCl, 9 g/day) failed to normalize the serum potassium (peaked at 2.3 mmol/L). As the level of serum creatine kinase (CK) was remarkably elevated(1268.4 u/L, normal range 50–200 u/L), the patient was transferred to our hospital. Upon admission, mild moon face and facial hyperpigmentation was noticed when compared with photos taken 2 years before. Oedema of the lower extremities and decreased muscle strength were also found during physical examination. Hyperpigmentation at interphalangeal joints and ecchymosis gradually developed during hospitalization.

**Fig. 1:**
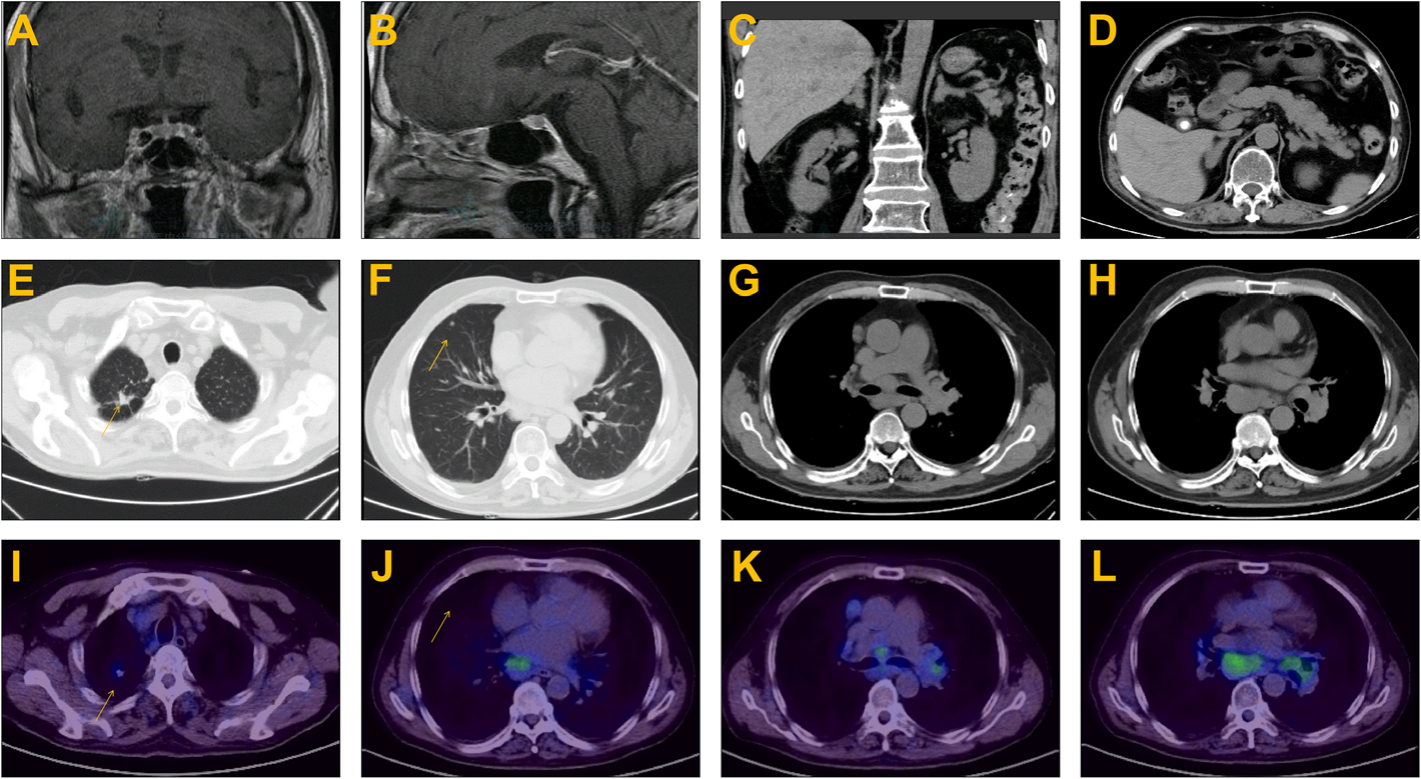
Imaging examinations. Pituitary MRI revealed no abnormalities (**a** and **b**). Abdominal CT showed diffuse enlargement of the bilateral adrenal glands (**c** and **d**). Chest CT indicated two nodules in the superior (**e**) and middle lobes of the right lung (**f**) and mediastinal lymphadenopathy (**g** and **h**). ^68^Ga PET/CT demonstrated high-density opacity in the superior lobe of the right lung with a slight abnormally increased uptake of ^68^Ga-DOTA-NOC (**i**). High-density nodules in the middle lobe of the right lung did not show abnormal uptake of ^68^Ga-DOTA-NOC (**j**, arrow). Multiple lymph nodes in the mediastinum and bilateral hili of the lung partially fused into a mass with abnormally increased uptake of ^68^Ga-DOTA-NOC (**k** and **l**)

**Fig. 2:**
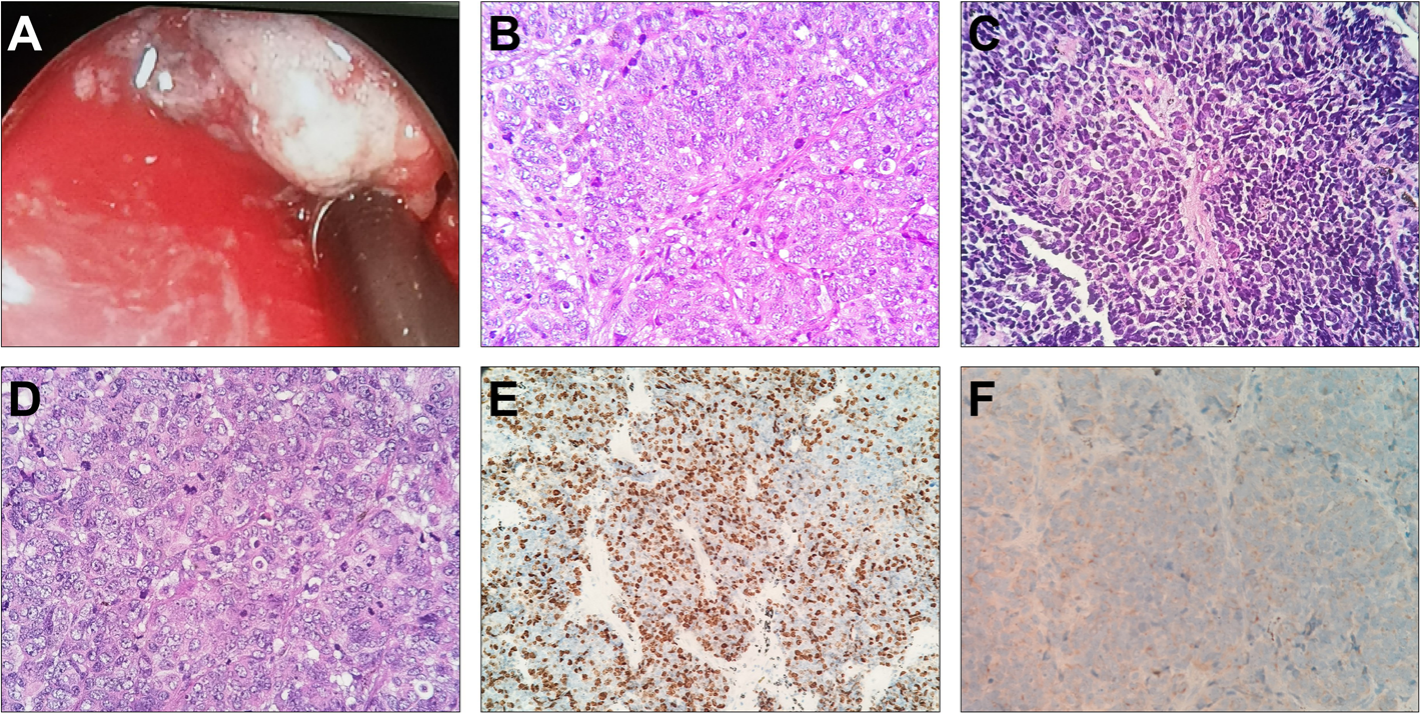
Mediastinoscopy revealed enlarged lymph nodes that were pale and friable (**a**). Large tumour cells with moderate to abundant cytoplasm and prominent nucleoli (**b**). Densely packed small tumour cells with scant cytoplasm, finely granular nuclear chromatin and absent nucleoli (**c**). Mitosis (**d**). Ki-67 index was 80 % (**e**). Immunohistochemical staining for ACTH (**f**). **b, c** and **d**, HE staining, 400×; **e** and **f**, 200×

The results of the laboratory test on admission and the function of the endocrine glands are summarized in Tables 1 and 2. Urinary potassium excretion was inappropriately increased (Table 1). The serum cortisol level was increased, and diurnal rhythm was lost. The level of serum cortisol at 8 am was > 50 µg/dL after the low-dose dexamethasone suppression test(LDDST), and the patient was diagnosed with CS. ACTH dependency was confirmed based on remarkably elevated ACTH levels (Table 2). Pituitary MR was normal (Fig. 1a and b). The serum cortisol level at 8 am before and after the high-dose dexamethasone suppression test (HDDST) was 212 µg/dL and 208 µg/dL (calculated with the measured value and dilution rate), respectively (Table 2). Although the above results strongly suggested EAS, bilateral inferior petrosal sinus sampling (BIPSS) was routinely arranged for verification, mainly for two reasons. First, false positive and false negative results occur in both HDDST and regular imaging(pituitary MR and chest CT). For example, the HDDST results for EAS and CD sometimes overlap. Some patients with EAS have coexisting pituitary adenomas that are not the source of ACTH. Second, in very few patients, CD is caused by hyperplasia instead of adenoma, resulting in a negative finding on pituitary MR. Then, BIPSS was successfully performed (PRL_left/_PRL_peripheral_ and PRL_right/_PRL_peripheral_ were 4.3 ± 0.5 and 2.5 ± 0.3, respectively). The left, right and peripheral ACTH levels were 789.9 ± 5.2 pg/mL, 773.6 ± 18.2 pg/mL and 726.5 ± 26.7 pg/mL (three samples for each), respectively, and the left, right and peripheral ACTH/PRL levels were 12.4 ± 1.4, 21.4 ± 2.4 and 49.1 ± 2.2, respectively. The diagnosis of EAS was made based on the HDDST and BIPSS results. Chest and abdominal CT as well as neck ultrasound were further arranged. Abdominal CT showed diffuse enlargement of the bilateral adrenal glands (Fig. 1c and d), and there were no abnormalities in the pancreas. Chest CT indicated two nodules in the superior and middle lobes of the right lung (Fig. 1e f) and prominent mediastinal lymphadenopathy (Fig. 1g h). Elevated tumour biomarkers, including carcinoembryonic antigen (CEA), cytokeratin 21 − 1 fragment CYFRA21-1 and neuron-specific enolase(NSE), were detected (Table 1). Thyroid ultrasound showed no abnormality. ^68^Ga-PET/CT suggested high-density opacity in the superior lobe of the right lung with a slight abnormally increased uptake of ^68^Ga-DOTA-NOC, fitting the characteristics of chronic infection (Fig. 1i). The high-density nodule in the middle lobe of the right lung was found without abnormal uptake of ^68^Ga-DOTA-NOC (Fig. 1j). Multiple lymph nodes in the left supraclavicular fossa, right root of the neck, mediastinum and bilateral hili of the lung were found partially fused into a mass with abnormal uptake of ^68^Ga-DOTA-NOC (Fig. 1k and l). Considering that the lymph nodes in the supraclavicular fossa and root of the neck might be distant metastases which is differ from the primary lesion, lymph nodes in the mediastinum and bilateral hili were preferable for biopsy. Therefore, mediastinoscopic lymph node biopsy was performed. The lymph nodes were pale and friable (Fig. 2a). The pathological diagnosis was small-cell and large-cell neuroendocrine carcinoma (LCNEC)according to the 2018 World Health Organization expert consensus proposal on the classification framework for neuroendocrine neoplasms [3] (Fig. 2b and c). Mitosis was approximately 80/10 HPF (Fig. 2d), and the Ki-67 index was 80 % (Fig. 2e). ACTH staining was positive (Fig. 2f).

**Table 1:** Laboratory findings of the patient on admission

Parameter	Patient’s value	Reference range
Blood routine		
WBC	11.19	3.5–9.5 × 10^9^/L
Neut%	10.53	1.8–6.3 × 10^9^/L
Hb	151	130–175 g/L
PLT	149	125–350 × 10^9^/L
Urine routine		
pH	7.5	5.5-8.0
Glu	-	-
Ket	-	-
Liver function		
ALT	52	9–50 U/L
AST	96	15–40 U/L
GGT	25	10–60 U/L
ALP	77	45–125 U/L
TBil	13.3	3.4–17.1 µmol/L
DBil	3.9	0-3.4 µmol/L
TP	50.5	65–85 g/L
ALB	30.6	40–55 g/L
Renal function		
BUN	5.91	3.9–9.5 mmol/L
Cr	64	57–111 µmol/L
UA	289	208–428 µmol/L
eGFR	99.27	> 90 mL/min/1.73m^2^
Blood gas analysis		
PH	7.58	7.35–7.45
PCO_2_	51	35–45 mmHg
PO_2_	63	80–100 mmHg
SB	40.8	22–27 mmol/L
BE	20.5	-3-+3 mmol/L
HCO_3_^-^	46.7	22–27 mmol/L
Electrolyte		
Urine potassium^*^	155.7	25–100 mmol/24 h
Synchronizing serum potassium	2.49	3.5–5.3 mmol/L
Urine sodium	296.4	130.0-260.0 mmol/24 h
Synchronizing serum sodium	142	137–147 mmol/L
Urine calcium	9.35	25.0–38.0 mmol/24 h
Synchronizing serum calcium	1.83	2.11–2.52 mmol/L
Calibration of serum calcium^#^	2.02	2.11–2.52 mmol/L
Urine phosphorus	24.68	32.3–38.4 mmol/24 h
Synchronizing serum phosphorus	0.74	0.85–1.51 mmol/L
FPG	7.4	3.9–6.1 mmol/L
HbA1c	5.7	4–6 %
OGTT		
Glucose-0 min	7.82	3.9–6.1 mmol/L
Glucose − 30 min	8.79	
Glucose − 60 min	9.41	
Glucose-120 min	7.95	< 7.8 mmol/L
Glucose − 180 min	6.94	
Insulin-0 min	151.5	5.0–25 mIU/L
Insulin-30 min	186.5	
Insulin-60 min	136.2	
Insulin-120 min	145.8	
Insulin-180 min	174.1	
Lipid		
CHO	3.57	3.1–5.69 mmol/L
TG	1.09	0.56–1.47 mmol/L
LDL	1.89	2.07–3.10 mmol/L
Coagulation function		
PTA	84 %	84-128 %
PT	14.6	11–14 s
TT	17.8	14–21 s
APTT	31.5	28-43.5 s
INR	1.11	0.94–1.3
FIB	2.08	2–4 g/L
FDP	0.98	0–5 mg/L
D-D	0.77	0–1.0 mg/L
Mb	741.3	0-146.9 ng/mL
TnT	0.067	0-0.014 ng/mL
Myocardial enzyme		
CK	695	50–310 U/L
CKMB	73	0–24 U/L
Pro-BNP	182.6	0-125 pg/mL
PCT	1.45	< 0.5 ng/mL
Tumour biomarkers		
CEA	4.930	0.00-3.40 g/mL
NSE	52.36	0.00-16.30 ng/mL
CYFRA21-1	8.750	0.00-3.30 ng/mL
CA19-9	44.11	0–39 U/mL
SCCA	0.77	< 1.5 ng/mL
Free-PSA	0.474	0–4 ng/mL
AFP	5.83	0–7 ng/mL
CA125	21.4	0–35 U/mL
CA72-4	1.6	0-9.8 U/mL

*WBC* white blood cell count, *Neut%* percentage of neutrophils, *Hb* haemoglobin, *PLT* platelet, *Glu* glucose, *Ket* ketone, *ALT* alanine aminotransferase, *AST* aspartate aminotransferase, *GGT* glutamyltranspeptidase, *TBil* total bilirubin, *DBil* direct bilirubin, *TP* total protein, *ALB* albumin, *BUN* blood urea nitrogen, *Cr* creatinine, *UA* uric acid, *eGFR* estimated glomerular filtration ratio, *FPG* fasting plasma glucose, *HbA1c* glycosylated haemoglobin, *OGTT* oral glucose tolerance test, *CHO* cholesterol, *TG* triglyceride, *LDL* low-density lipoprotein cholesterol, *PCO*_2_ partial pressure of carbon dioxide, *PO*_2_ partial pressure of oxygen, *SB* standard bicarbonate, *BE* base excess, *HCO3*^-^ bicarbonate, *Mb* myoglobin, *TnT* troponin T, *pro-BNP* pro-brain natriuretic peptide, *PCT* procalcitonin, *CK* creatine kinase, *CKMB* creatine kinase isoenzyme, *PTA* prothrombin time activity, *PT* prothrombin time, *TT *thrombin time, *APTT* activated partial thrombin time, *INR *international normalized ratio, *FIB* fibrinogen, *FDP* fibrin degradation product, *D-D* D dimer, *CEA* carcinoembryonic antigen, *NSE* neuron-specific enolase, *CYFRA21-1* cytokeratin 21 − 1 fragment, *CA19-9* carbohydrate antigen 19 − 9, *SCCA* squamous cell carcinoma antigen, *PSA* prostate specific antigen, *AFP* alpha foetal protein, *CA125* carbohydrate antigen 125, *CA72-4* carbohydrate antigen 72 − 4. *, the criteria of inappropriate increased urinary potassium excretion are urine potassium >25mmol/24h when serum potassium <3.5mmol/L or urine potassium >20mmol/24h when serum potassium <3.0mmol/L. ^#^, Calcium_calibrated_ =Calcium_measured_+(40-serum albumin) ×0.02

**Table 2:** Function of the endocrine glands

Parameter	Patient’s value	Reference range
ACTH-COR		
ACTH-8:00	647.2	7.2–63.3 pg/mL
COR-8:00	> 50	5–28 µg/dL
COR-16:00	> 50	µg/dL
COR-24:00	> 50	µg/dL
Urine 17-KS (24 h)	221.48	20.8–76.3 µmol/24 h
Urine 17-OHCS (24 h)	482.5	8.32–33.2 µmol/24 h
LDDST		
COR-before	> 50	µg/dL
COR-after	> 50	µg/dL
HDDST^a^		
COR-before	212	µg/dL
COR-after	208	µg/dL
BIPSS		
Left		
PRL-1	56.27	ng/mL
PRL-2	67.00	ng/mL
PRL-3	69.01	ng/mL
ACTH-1	787.4	pg/mL
ACTH-2	795.9	pg/mL
ACTH-3	786.4	pg/mL
Right		
PRL-1	33.91	ng/mL
PRL-2	40.62	ng/mL
PRL-3	34.53	ng/mL
ACTH-1	794.6	pg/mL
ACTH-2	762.6	pg/mL
ACTH-3	763.6	pg/mL
Peripheral		
PRL-1	15.01	ng/mL
PRL-2	14.4	ng/mL
PRL-3	14.96	ng/mL
ACTH-1	751	pg/mL
ACTH-2	730.5	pg/mL
ACTH-3	698	pg/mL
Renin-Angiotensin-Aldosterone		
Renin	7.88	4–24 pg/mL
Angiotensin	45.29	25–129 pg/mL
Aldosterone	75.15	10–160 pg/mL
ARR	9.54	0–45
Gonadal hormone		
E2	117.9	28–156 pmol/L
Prog	5.65	0.159-0.5 nmol/L
PRL	23.24	4.04–15.2 ng/mL
LH	4.24	1.7–8.6 mIU/mL
FSH	6.4	1.5–12.4 mIU/mL
T	4.26	6.68–25.7 nmol/L
DHEAS	8.48	1.4–8.01 µmol/L
Thyroid function		
Free T3	3.23	2.91–9.08 pmol/L
Free T4	15.00	9.05–25.5 pmol/L
T3	< 0.05	0.78–2.20 ng/mL
T4	3.21	4.2–13.5 µg/dL
TSH	0.56	0.25-5 µIU/mL
TPOAb	< 15	< 15 U/mL
Parathyroid function		
PTH	165.7	15–65 pg/mL
25(OH)D_3_	13.3	20–40 ng/mL
N-OST	9.0	14–46 ng/mL
P INP	19.16	9.06–76.24 ng/mL

*ACTH* adrenocorticotropic hormone, *COR* cortisol, *17-OH* 17-ketosteroids, 17-hydroxycorticosteroids, *LDDST* low-dose dexamethasone suppression test, *HDDST* high-dose dexamethasone suppression test, *BIPSS* bilateral inferior petrosal sinus sampling, *E2* oestradiol, *Prog* progesterone, *PRL* prolactin, *LH* luteinizing hormone, *FSH* follicle-stimulating hormone, *T* testosterone, *DHEAS* dehydroepiandrosterone sulfate, *ARR* the aldosterone and active renin ratio, *TSH* thyroid-stimulating hormone, *T3* triiodothyronine, *T4 *thyroxine, *TPOAb* thyroid peroxidase antibody, *PTH* parathyroid hormone, *25(OH)D*_3_ 25-hydroxyvitamin D3, *N-OST* N-osteocalcin, *PINP* procollagen IN-terminal peptide

^a^calculated with the measured value and dilution rate

**Fig. 3:**
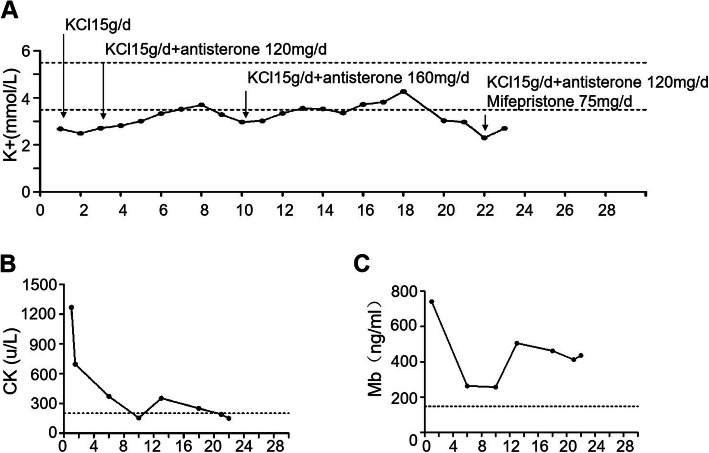
Serum potassium level and corresponding treatment (**a**). Fluctuation of serum CK and Mb levels (**b** and **c**). The space between dashed lines (**a**) or between the dashed line and the x-axis (**b** and **c**) is the normal range

The fasting plasma glucose (FPG) level was 7.4 mmol/L, and the glycosylated haemoglobin(HbA1c) level was 5.7 % on admission. During hospitalization, the FPG level gradually increased. Diagnosis of diabetes was made, and insulin therapy was initiated. Bone mineral density indicated low bone mass. Blood pressure, lipid profile, and coagulation function were generally normal.No thrombus was found by colour Doppler ultrasound in the arteries or veins of the lower extremities. On admission, remarkably elevated levels of serum CK and myohemoglobin (Mb)were noticed with slightly increased creatine kinase isoenzyme(CKMB) (Table 1). ECG was generally normal. Based on the above results and the even higher CK level (higher than 5 times the upper limit) at the local hospital, the diagnosis of RM was made, and fluid infusion was applied. The serum potassium level was 2.49 mmol/L on admission, and potassium supplementation (KCl, 15 g/d) was initiated. After the assessment of renin, angiotensin and aldosterone, antisterone was prescribed at a daily dose of 120–160 mg. The CK and Mb levels decreased gradually as the serum potassium level increased. On day 10, a saline and glucose solution was applied after the enhanced pituitary MR and chest CT to prevent acute kidney injury, and transient decline in the serum potassium level and re-elevation of the CK and Mb levels occurred (Fig. 3a, b and c). On admission, an increased troponin T level, a decreased serum calcium level and an increased parathyroid hormone(PTH)level were also observed and were gradually relieved with the improvement in RM.

After discussions among specialists from multiple disciplines (pathology, respiratory medicine, oncology, anaesthesiology, urology, thoracic surgery and endocrinology department), the urologists and anaesthetists considered it very risky for the patient to undergo bilateral adrenalectomybased on hypokalaemia, infection and general status. Additionally, aggravation of the condition was more related to malignancy than hypercortisolaemia, especially after the application of mifepristone. As the priority was determined to be to treat neuroendocrine carcinoma, which was unresectable, the patient was transferred to the oncology department to receive chemotherapy. In addition, mifepristone was applied at an initiating dosage of 75 mg per day and a maintaining dosage of 150 mg per day. The patient received one cycle of chemotherapy with the EP regimen (etoposide 100 mg iv drip days 1–3 and cisplatin days 1–3) but could not tolerate subsequent chemotherapy. He died of dyscrasia within 1 month.

## Discussion and conclusion

We reported a representative case of EAS caused by a very aggressive malignancy, with atypical manifestation of CS and no opportunity for radical resection at diagnosis. As a rare cause of CS, EAS has always been a clinical challenge. First, EAS often has an insidious onset. Typical signs of CS, such as moon face and central obesity, are often absent from the influence of cachexia, leading to delayed diagnosis. Second, some tumours are highly malignant and progress rapidly, and diagnosis and treatment are urgently needed. The diagnosis from ACTH-dependent CS to the specific causes was roughly divided into two steps. The first step is to distinguish whether the lesion is located in the centre (pituitary or hypothalamus) or the periphery. The amplitude of ACTH and the HDDST, pituitary MR, chest and abdomen CT results can be suggestive. However, as the incidence of incidental pituitary adenomas is as high as 10 %, identification of a pituitary adenoma does not always guarantee the identification of the responsible lesion. Moreover, the ACTH levels and HDDST results of some peripheral carcinoid and pituitary adenomas overlap. Some tumour biomarkers are not specific and increase in chronic inflammation. With the combination of HDDST and BIPSS, 95 % of the cases can be distinguished [2, 4, 5]. However, due to the technical skills required to perform BIPSS and vascular variation, this procedure sometimes fails, and the results can be ambiguous in certain cases. The ultimate challenge is the localization of the tumour. EAS is most frequently caused by lung or mediastinal carcinoid and small-cell lung cancer(SCLC). The less common causes include insulinoma, medullary thyroid cancer, gastrointestinal neuroendocrine tumour and pheochromocytoma [2]. Therefore, chest CT should be applied first, and other imaging modalities, including abdominal CT and thyroid ultrasound, could be arranged. One difficulty is that the high incidence/detection rate of pulmonary nodules results in more than one suspected lesion in some cases. As in this patient, two nodules were found in the right lung, while enlarged lymph nodes were found in the mediastinum and both hilar regions. PET/CT can be further applied if CT fails. Specifically, ^68^Ga PET/CT is effective in locating pulmonary neuroendocrine tumours, while 18-F FDP is suitable for malignancies known or speculated to be more aggressive. For ^68^Ga and 18-F FDP PET/CT-negative cases, somatostatin imaging can be adopted, but persistent hypercorticosteraemia may suppress the expression of the somatostatin subtype 2 receptor and cause false-negative results in some cases [6]. Dopamine receptor PET/CT is used mainly to locate tumours with co-secretion of catecholamine and ACTH. For cases with negative functional imaging, segmental body vein blood sampling can theoretically be used for localization but is of limited value for adjacent lesions sharing the same drainage vein. Fibrobronchoscopic and percutaneous transthoracic biopsy can be applied to verify the suspected lesions in the lung, and endobronchial ultrasound and mediastinoscopy biopsies can be applied for those in the mediastinum. For our patient, either endobronchial ultrasound or mediastinoscopy biopsies were feasible, and mediastinoscopy biopsy was ultimately opted to obtain sufficient tissue and to facilitate haemostasis under direct vision in case of intraoperative bleeding.

The ultimate cure for patients with EAS is radical resection of the malignancy, which was not feasible for our patient who had multiple metastases. LCNEC and small-cell carcinoma are both high-grade neuroendocrine carcinomas. Early surgical resection is the first-line therapy for LCNEC, and the 5-year and 10-year survival rates are approximately 30 and 10 %, respectively. Most patients with small-cell carcinoma require chemotherapy or a combination of chemotherapy and radiotherapy, and the 5-year and 10-year survival rates are approximately 10 % and less than 5 %, respectively [7]. For EAS patients with unresectable lesions, medicines have been reported to be effective in normalizing cortisol levels in 83.3 %(64.9–96.8) of patients [8]. Since neither mitotane nor cabergoline are marketed in China, the glucocorticoid receptor antagonist mifepristone is also used for palliative therapy.

This patient had RM caused by extreme hypokalaemia, a rare complication of EAS. There are diverse causes of rhabdomyolysis. Patients with CS are susceptible to infection due to immunodeficiency caused by hypercortisolaemia, which is a common aetiology for RM. Disturbances of electrolytes such as hyponatraemia, hypokalaemia and hypophosphataemia should also be considered in the aetiological diagnosis of RM [9]. For the former, some tumours causing EAS may secrete peptides causing SIADH. Pulmonary infection is also common in patients with EAS and may also result in SIADH [10, 11]. Our patient did not exhibit the manifestations of SIADH. Both the serum sodium level and the osmotic pressure were generally normal during hospitalization, ruling out SIADH. This patient had mild hypophosphataemia. However, based on the severity of hypophosphatemia and the reversing trend of the levels of CK and K^+^, we considered hypokalaemia instead of hypophosphatemia to result in RM.In patients with CS, extra cortisol binds to mineralocorticoid receptors and leads to hypokalaemia. Some medications, such as diuretics and mifepristone, contribute to aggravation. Hypokalaemia in patients with EAS or adrenocortical carcinoma(ACC) is often intractable due to the extremely high cortisol level. K^+^ regulates blood flow in muscle tissue by dilating vessels. As skeletal muscles consume large amounts of oxygen, hypokalaemia impairs blood supply and causes ischaemic damage. Hypokalaemia also increases the permeability of the myolemma by disturbing transmembrane ion transport [12]. The severity of muscle injury is closely related to plasma potassium levels. Myasthenia and myalgia may be reported when the K^+^level is below 3.0 mmol/L, elevation of myoenzymes may occur when the K^+^level is below 2.5 mmol/L, and RM might occur when the K^+^level is below 2.0 mmol/L [9, 12].

An interesting issue is that RM is reported far more frequently in primary aldosteronism (PA), although PA and CS are both related to severe hypokalaemia. One explanation is the very low incidence of EAS and ACC. Another possible mechanism is that hypokalaemia-induced RM is caused by the imbalance of oxygen supply and consumption. Patients with EAS and ACC usually have significant protein catabolism and muscle loss due to extreme hypercorticosteraemia. Many of them also have restricted physical activities due to pathological fracture or cachexia. As oxygen consumption is determined by muscle content and activity status (resting or exercising), patients with CS have a lower oxygen requirement. In contrast, muscle content and physical activity are generally normal in patients with PA, making them more vulnerable to impaired blood and oxygen supply caused by hypokalaemia.

Clinical manifestations of RM vary greatly. The typical triad of myasthenia, myalgia and coloured urine occurs in less than 10 % of patients, while more than 50 % of patients have no symptoms or signs [12–15]. Endocrine disease-related RM is even more insidious. Muscle enzymes should be measured, and RM should be considered in patients with CS or PA when the serum potassium level is below 2.0 mmol/l. From a monitoring aspect, since Mb can be eliminated within 24 h from serum, its re-elevation suggests new onset of muscle damage [16]. The treatment of RM should be focused on fluid replacement and the consequent adjustment of potassium supplementation. For CS patients with RM, hypokalaemia and metabolic alkalosis to some extent alleviate the hyperkalaemia and hyperlactacidaemia caused by RM, and complications, including acute renal injury and arrhythmia, are rare. It should also be noted that with the destruction of muscle cells, a large amount of phosphate is released that binds to calcium ions and accumulates in the damaged muscle, resulting in hypocalcaemia [17], as was observed in this patient. PTH elevation is secondary to hypocalcaemia, which does not need to be treated. Calcium supplementation is also unnecessary and might cause ectopic calcification in muscle tissue [18].

In summary, RM is a rare complication of EAS with atypical clinical manifestations. Serum potassium levels should be vigilantly monitored to avoid RM in EAS.

## Data Availability

All relevant data supporting the conclusions of this article are included within the article.

## Abbreviations

CS: Cushing’s syndrome
EAS: Ectopic adrenocorticotropic hormonesyndrome
ACTH: Adrenocorticotropic hormone
RM: Rhabdomyolysis
CK: Creatine kinase
LDDST: Low-dose dexamethasone suppression test
MR: Magnetic resonance
HDDST: High-dose dexamethasone suppression test
BIPSS: Bilateral inferior petrosal sinus sampling
PRL: Prolactin
CT: Computed tomography
CEA: Carcinoembryonic antigen
CYFRA21-1: Cytokeratin 21 − 1 fragment
NSE: Neuron specific enolase
LCNEC: Large-cell neuroendocrine carcinoma
WHO: World Health Organization
FPG: Fasting plasma glucose
HbA1c: Glycosylated haemoglobin
Mb: Myohemoglobin
CKMB: Creatine kinase isoenzyme
ECG: Electrocardiogram
PTH: Parathyroid hormone
SIADH: Syndrome of inappropriate antidiuretic hormone
ACC: Adrenocortical carcinoma
PA: Primary aldosteronism
